# Predictors of late diagnosis of prostate cancer among men of African ancestry receiving care at urology clinic, Federal Medical Centre, Abeokuta

**DOI:** 10.3332/ecancer.2025.1917

**Published:** 2025-05-29

**Authors:** Shola Blessing Olorunniyi, Chidiebere Ndukwe Ogo

**Affiliations:** Urology Unit, Department of Surgery, Federal Medical Centre, PMB 3031, Sapon Post Office, Abeokuta, Ogun State, Nigeria

**Keywords:** health belief model, late diagnosis, prostate cancer, Sub-Saharan Africa

## Abstract

Prostate cancer (CaP) is a significant global health challenge, ranking as a leading cause of cancer mortality among men, particularly in Sub-Saharan Africa. In Nigeria, CaP accounts for 37.5% of new cancer cases and a high mortality rate, largely attributed to late-stage diagnoses. While early detection through screening methods such as digital rectal examination (DRE) and prostate-specific antigen (PSA) testing can improve survival outcomes, barriers persist, especially among men of African ancestry who are at higher risk from age 40. This study explores the reasons behind delayed screening and late diagnosis in Sub-Saharan Africa, identifying barriers using the health belief model as a framework; hence, the research explored key constructs: perceived susceptibility, severity, benefits, barriers (cues to action) and self-efficacy. This cross-sectional study specifically examines these predictors among adult males attending a urology clinic in Abeokuta, Nigeria. A simple random sampling technique was used to recruit 128 study participants. The study found that, despite empirical evidence highlighting increased susceptibility to CaP from the age of 40, most participants perceived themselves as not at risk, indicating a significant lack of awareness. This low perceived susceptibility negatively impacts health-seeking behaviours, including early screening. Participants generally acknowledged the severity of CaP, which should ideally motivate preventive actions. However, many found decision-making about screening and undertaking annual DRE or PSA tests challenging, reflecting low self-efficacy. While participants recognised the benefits of preventive measures, barriers such as embarrassment, fear of pain and lack of physician recommendations were reported. Notably, the absence of screening recommendations by healthcare providers emerged as a significant gap, despite guidelines advocating early screening for men of African ancestry. These findings underscore the need for targeted interventions to raise awareness, enhance self-efficacy, address procedural concerns and encourage proactive physician involvement in recommending screenings to mitigate the high prevalence of late-stage diagnoses of CaP.

## Background

The prostate gland is a vital component of the male reproductive system responsible for producing prostatic fluid. This fluid plays a crucial role in nourishing and maintaining sperm, which is released during ejaculation [1].

Prostate cancer (CaP) is a major global health concern and ranks among the leading causes of cancer-related deaths in men. Data from GLOBOCAN 2022 indicates that CaP is the leading cause of cancer mortality among Nigerian men, with 18,019 new cases (37.5%) and 11,443 deaths reported. Similarly, across Africa, CaP accounts for 103,050 new cases (20.4%) and 55,744 deaths. On a global scale, it was responsible for 1,467,854 new cases and 397,430 deaths [2].

Despite its severity, CaP can be effectively managed and even cured if detected early [3]. However, in Sub-Saharan Africa, most men are diagnosed at advanced stages of the disease, largely due to delayed presentation of the disease to the hospital [4]. This late diagnosis significantly contributes to the high mortality rate associated with CaP, as survival rates diminish when the disease progresses to advanced stages [3]. Consequently, early screening has been identified as a key secondary prevention strategy to reduce mortality rates [3].

Furthermore, the risk of having CaP is higher among adult males [5]. Men of African ancestry are at higher risk of the disease from the age of 40, while men of European ancestry are at higher risk from 50 years of age [6]. Consequently, men of African ancestry are advised to begin screening for CaP from the age of 40 years. Common screening methods include digital rectal examination (DRE), prostate-specific antigen (PSA) testing, prostate biopsies and imaging [7].

CaP risk factors can be categorised as modifiable and non-modifiable. Modifiable factors include lifestyle choices such as smoking, high-fat diets, tobacco use and excessive alcohol consumption [8]. Avoiding these risk factors serves as a primary prevention measure. Non-modifiable factors, such as age, race and family history, necessitate secondary prevention through early screening, particularly for men with a family history of the disease [8].

Additionally, in the natural progression of CaP, early detection through methods such as DRE, PSA testing and biopsies can halt disease progression or lead to a complete cure, especially when caught in the early stages (stage one or two) [3]. Secondary prevention measures, including hormonal therapy, radiation and surgery, are effective in managing the disease at these stages [9]. In contrast, when CaP advances to later stages (stage three or four) and metastasizes to other parts of the body, tertiary prevention strategies such as chemotherapy or castration surgery may be required [9]. These approaches aim to manage the disease, limit metastasis and prevent fatalities while addressing associated disabilities.

Therefore, this study was conducted to examine the reasons why most men of African ancestry in the sub-Saharan Africa region were not getting screened early for CaP, why most men with the disease are diagnosed late and the barriers involved in this trend – using the health belief model (HBM).

## Aim

The general aim of this study is to examine why adult males, age 40 and above do not begin early/frequent screening for CaP, as a means of secondary prevention of CaP, to ensure early diagnosis of the disease. This aim was operationalised by the specific objective of assessing the barriers to early screening among adult males, who are at risk of developing the disease; assessing their perceived susceptibility to the disease; perceived severity; cues to action and self-efficacy.

Consequently, the HBM was employed in this process. Favourably, the development of the HBM was particularly for researches that elucidate preventive health practices/behaviour [10].

The HBM can be used to explain and predict individual health behaviours, and in this study, it was used to examine the predictors’ of late prostate diagnosis.

The HBM explains and predicts individual health behaviours using a number of elements, which are: **Perceived susceptibility** – the individual’s perceived threat to having the disease; in this case CaP; **Perceived severity** – the individual belief of the consequences of having the disease; **Perceived benefits** – the individuals understanding of the potential benefits to action; in this case benefit of early/regular CaP screening; **Cues to action** – the individual’s perceived barriers to action [i.e., barriers to cancer screening] and **Self-efficacy** – the individual’s confidence in their ability to succeed in the positive health behaviour and actions to be taken in terms of preventive measures (i.e., early/regular screening) [11].

Hugosson *et al* [12] was able to affirm that early PSA testing mitigates CaP mortality significantly and also reduces the morbidity rate. Meanwhile, Ugochukwu *et al* [13] stated that the primary barriers to screening included fear of a positive result, lack of awareness and financial constraints. Additionally, participants expressed a preference for male physicians during a DRE. In their research on CaP screening [13], while 54.9% of participants demonstrated limited knowledge about CaP and its screening methods, 65.7% displayed positive attitudes toward screening; however, only 21.2% had undergone screening.

Seraphin *et al* [4] found that the rate of late-stage presentation by Sub-Sarahan African men with prostatic tumour was high and consequently associated with poor outcomes and low survival rates of the disease [4].

Therefore, using the constructs in the HBM – perceived susceptibility, perceived severity, perceived benefits, cues to action and self-efficacy, this study was able to examine predictors’ of late presentation of CaP among adult males, receiving care in the urology clinic at Federal Medical Centre, Abeokuta, Ogun State, Nigeria.

## Methods

### Design

This study employed the cross-sectional study design in elucidating the problem phenomenon.

### Setting

This study was conducted in the urology clinics at Federal Medical Centre Abeokuta, located in the Abeokuta-South local government area of Ogun State, Nigeria, Sub-Sahara Africa.

### Sampling technique and eligibility criteria

Participants were selected using the simple random, probability sampling technique, from a pool of non-CaP patients, sampled using the purposive sampling technique.

The inclusion criteria for this study are: adult males of African ancestry (blacks), above 40 years of age and eligible patients who gave informed consent; this age range was chosen based on evidence indicating an increased risk of CaP among men of African ancestry from the age of 40 [6, 14]. On the other hand, the exclusion criteria are histologically diagnosed CaP patients and eligible participants who are critically ill and have significant cognitive impairment.

### Sample size

The computed sample size for this study was 123.3. At the end of data collection, 128 participants were recruited with a questionnaire completed.

The sample size for the study was determined using a prevalence rate of 8.8%, which was derived from a previous study conducted in Nigeria [15].

Using the formula for sample size determination for a single proportion:


$$n\;=\;\frac{Z^{2}*P*(1-P)}{E^{2}}$$

where:

*Z* = 1.96 (*z*-score for a 95% confidence level),

*P* = 0.088 (prevalence of CaP from the cited study),

*E* = 0.05 (margin of error).

The calculated sample size was approximately 123.3. To account for potential non-responses and inconsistencies in responses, we adjusted this value upwards to 128 participants to ensure a sufficient number of valid responses.

### Ethical approval

This study obtained ethical clearance and approval from the Health Research Ethics Committee at Federal Medical Centre, Abeokuta. Written consent was acquired from study participants.

### Instrument

The instrument of data collection was developed based on the HBM, the items in each variable (Perceived severity, perceived susceptibility, self-efficacy, perceived benefit and cues to action) measured the HBM constructs.

Study participants provided responses based on the variables in the HBM; perceived susceptibility, perceived severity, perceived benefits, cues to action and self-efficacy – on a Likert scale measure.

Data collected were imputed into Statistical Product and Service Solution (SPSS) and analysed using SPSS version 26. The Cronbach Alpha analysis was used for the reliability test of the instrument of data collection used for the study. Demographic responses were coded into rating scales to enable standard measurement. Descriptive statistics was conducted to examine the frequencies, percentages and mean of the responses for each variable item in the HBM structured questionnaire.

Demographic data showed information on place of residence, ethnicity, age, family history, level of education and occupation.

The ***perceived severity*** variable contained items such as how scared they are of CaP and their perception of how CaP would change their life if they had it. It consisted of five items with the Likert-type 5-response options (Strongly agree, agree, neither agree nor disagree (Neutral), disagree or strongly disagree; with one being the least and five being the highest). The result of the Cronbach Alpha analysis of the test of reliability for these items was a good score of .828.

This study measured the ***perceived susceptibility*** with four items, asking questions of what they perceive as their chances of getting CaP; the four items were measured on a Likert-type 5-response options (Strongly agree, agree, neither agree nor disagree (Neutral), disagree or strongly disagree; with one being the least and five being the highest). The result of the Cronbach Alpha analysis of the test of reliability for these items was 0.771.

The ***self-efficacy*** scale consisted of three items which had a Cronbach Alpha of test of reliability score of 0.761 and this variable measured the individual’s confidence in their ability to make the decision of having a CaP screening yearly. The three items were measured on a Likert-type 5-response option (Very easy, easy, neither easy nor difficult (Neutral), difficult or very difficult; with one being the least and five being the highest).

The ***perceived benefit*** variable included questions on the positive outcomes of CaP screening like early detection and peace of mind. It consisted of four items using the Likert-type 5 response options (Strongly agree, agree, neither agree nor disagree (Neutral), disagree or strongly disagree; with one being the least and five being the highest). The result of the Cronbach Alpha analysis of the test of reliability for these items was 0.933.

The ***cues to action*** variable asked questions to examine the barriers to CaP screening like was it because it was not recommended, was it forgetfulness, fear, busy schedule, DRE embarrassment, financial barrier or ignorance. It consisted of nine items using the Likert-type 5 response options (Strongly agree, agree, neither agree nor disagree (Neutral), disagree or strongly disagree; with one being the least and five being the highest). The result of the Cronbach Alpha analysis for the test of reliability for these items was a good score of 0.804.

### Decision rule

The weighted average or ground mean (cumulative mean/No. of items) of each construct was used as the decision rule to determine the perception level of the individual items. An item means, lower than the weighted average indicates low perception, while an item means higher than the weighted average indicates high perception in each construct.

### Data collection

Research data were collected using a pencil and paper questionnaire; the questionnaires were administered by the researcher to the participants using the interview method.

### Theoretical underpinning

**Figure 1.**
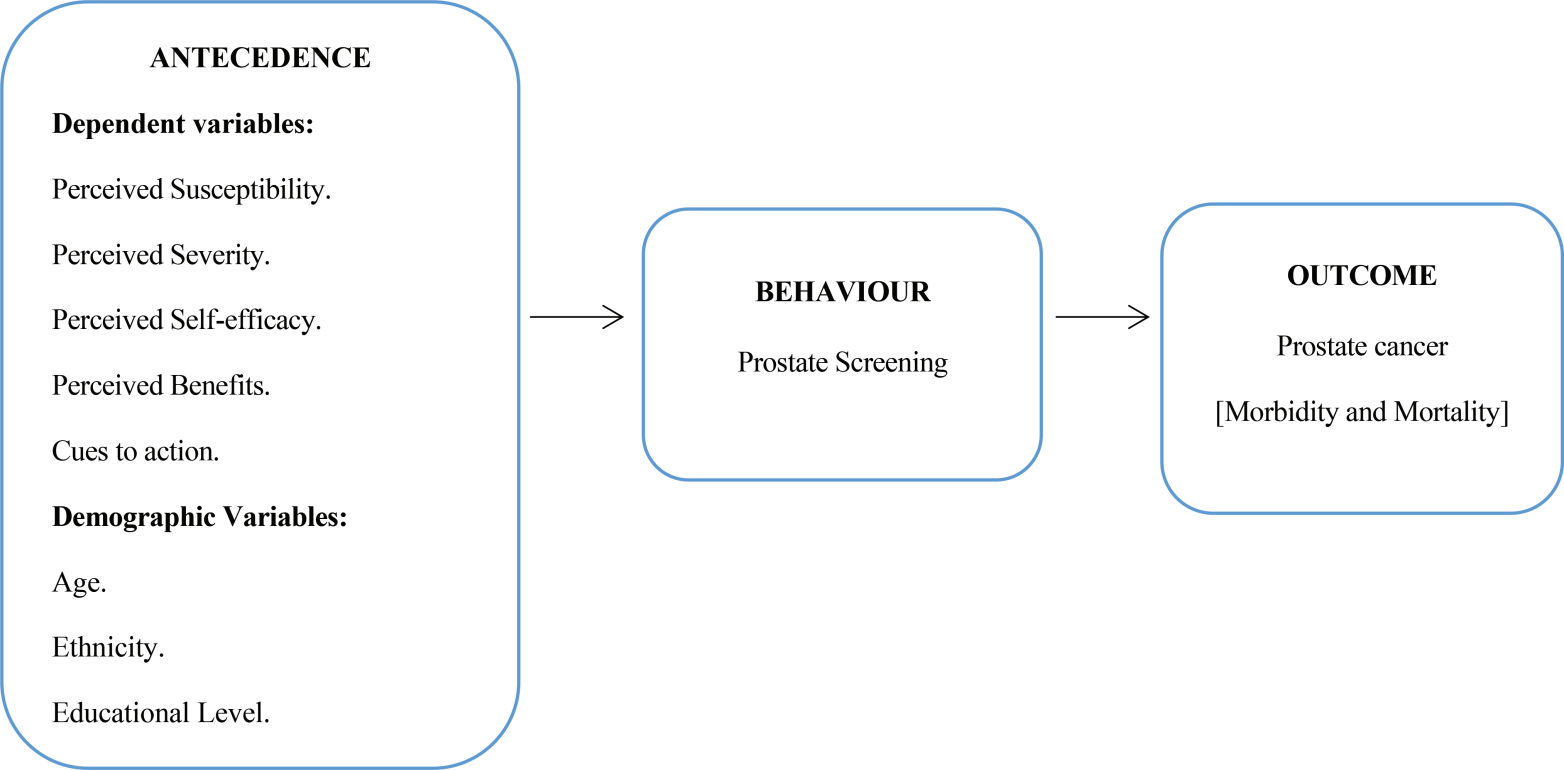
Conceptual framework, conceptualised adopting the HBM, showing the linkage between the antecedent factors, behavioural factors and consequences (outcome).

This study adopted the HBM, to build the science describing the predictors of late presentation of CaP, among adult males. The model is comprised of five constructs: Perceived susceptibility, perceived severity, perceived benefits, cues to action and self-efficacy (Figure 1).

*Perceived susceptibility* is the individual’s perceived threat to having the disease; in this study, we assessed the study participants’ perceived susceptibility to CaP. *Perceived severity* is the individual’s belief of the consequences of having the disease; for this study, we assessed the participants’ perceived severity of CaP disease. *Perceived benefits* is the understanding of the potential benefits to action; for this research, we assessed their perception of the benefits of early/regular CaP screening. *Cues to action* are the individual’s perceived barriers to action; in this study, we assessed the barriers to CaP screening. *Self-efficacy* is the individual’s confidence in their ability to succeed in the positive health behaviour and actions to be taken in terms of preventive measures; therefore, we assessed the participants’ self-efficacy in terms of early/regular CaP screening.

## Results

### Data analysis

The purpose of this study was to examine the predictors of late diagnosis of CaP among adult males in Abeokuta, Ogun State, Nigeria.

Hence, the study population consisted of 128 adult males, 62.5% were between the ages of 40–50 years, 25% were between 51 and 60 years and 12.5% were between 61 and 70 years. The study site was located in southwestern Nigeria, a predominantly Yoruba ethnic group community; consequently, the major ethnic group was Yoruba (75%), while other ethnic groups, including Igbo and Hausa, accounted for the remaining 25% cumulatively. Meanwhile, 9.38% had a primary level of education, and 78.1% and 12.5% had secondary and tertiary levels of education, respectively. Additionally, 25% were retired, while 12.5%, 50% and 12.5% were in civil service, private sector and business owners, respectively (Table 1).

### Perceived susceptibility

The data analysis showed that, when respondents were asked ‘my chances of getting CaP are great,’ the majority (62.5%) **strongly disagreed.** Also, when asked ‘there is a good possibility that I will get CaP,’ majority of the respondents (50%) **strongly disagreed**. Additionally, when asked ‘I am not at risk for CaP,’ the majority of the respondents (50%) **strongly agreed**. Meanwhile, when asked ‘there is no chance that I will get CaP,’ the majority of the respondents (62.5%) **strongly agreed** (Table 2).

### Perceived severity

The data analysis showed that, when respondents were asked ‘if I had CaP my whole life would change,’ the majority (37.5%) **strongly agreed/agreed**. Similarly, when asked ‘getting CaP would significantly affect my life,’ the majority of the respondents (37.7%) **agreed**. Meanwhile, when asked ‘I get very scared when I think of CaP,’ the majority of the respondents (50%) **disagreed**. Additionally, when asked ‘getting CaP would significantly affect my manhood,’ the majority of the respondents (25%) **strongly disagreed** (Table 3).

### Perceived self-efficacy

The data analysis showed that, when respondents were asked about ‘making a decision about CaP screening,’ majority (75%) said it was **difficult**. Meanwhile, when asked about ‘having a DRE every year,’ the majority of the respondents (37.7%) said it was **difficult/very difficult**. Furthermore, when asked about ‘giving blood sample for serum PSA test every year,’ the majority of the respondents (62.5%) said it was **difficult** (Table 4).

### Perceived benefits

The data analysis showed that, when respondents were asked *‘preventing CaP through activities such as eating right, taking supplements and exercising will save my life*,’ the majority (75%) **strongly agreed**. Also, when asked *‘preventing CaP through activities such as eating right, taking supplements, and exercising will help me to prevent other diseases*,’ the majority of the respondents (62.5%) **strongly agreed**. Meanwhile, when asked *‘screening for CaP every year will allow me to detect the disease early and get appropriate treatment on time,’* the majority of the respondents (50%) **strongly agreed**. Additionally, when asked ‘*getting screened for CaP every year will give me peace of mind*,’ the majority of the respondents (50%) **strongly agreed** (Table 5).

### Perceived cues to action

The data analysis showed that, when respondents were asked ‘I do not get tested for CaP because my doctor has not recommended this test,’ the majority (37.5%) **strongly agreed**. Meanwhile, when asked ‘I do not get tested for CaP because I forgot to do it,’ the majority of the respondents (62.5%) **disagreed**. Additionally, when asked ‘I do not get tested for CaP because I fear finding cancer,’ the majority of the respondents (50%) **disagreed**. Furthermore, when asked ‘I have more important things to do that stops me from getting tested for CaP,’ the majority of the respondents (25%) **disagreed/strongly disagreed**. Moreover, when asked ‘I am too busy to be tested for CaP,’ the majority of the respondents (37.5%) **disagreed.** Additionally, when asked ‘I am embarrassed to be tested for CaP,’ the majority of the respondents (50%) **agreed**. Meanwhile, when asked ‘I do not get tested for CaP because I cannot afford the test,’ the majority of the respondents (37.5%) **disagreed**. However, when asked ‘I do not get tested for CaP because I am afraid of experiencing pain and discomfort,’ the majority of the respondents (50%) **agreed.** Meanwhile, when asked ‘I do not get tested for CaP because I am not of screening age,’ a cumulative majority of the respondents (62.5%) **disagreed/strongly disagreed** (Table 6).

## Discussion

The findings of this study highlight the predictors of late CaP diagnosis among adult males in Abeokuta, Ogun State, Nigeria. A key observation from the study is the low perceived susceptibility to CaP among participants. The majority of respondents strongly disagreed with statements suggesting that their chances of developing CaP were high, and many believed they were not at risk. This finding aligns with previous studies that have reported low awareness and risk perception of CaP among Nigerian men [16, 17]. Lack of perceived susceptibility has been associated with reduced engagement in screening behaviours, leading to delayed diagnosis and poor health outcomes [18].

In terms of perceived severity, while many respondents acknowledged that CaP could significantly impact their lives, a substantial proportion did not express fear about the disease. This lack of concern may contribute to the reluctance to seek early screening and diagnosis. Prior research has demonstrated that individuals who perceive disease as severe are more likely to adopt preventive health behaviours [19]. Therefore, the observed moderate perception of severity suggests a need for targeted education to emphasise the serious consequences of late-stage CaP.

Perceived self-efficacy was another significant factor influencing screening behaviour. A high proportion of respondents found it difficult to make decisions about CaP screening, particularly regarding the DRE. Studies have shown that low self-efficacy regarding CaP screening is a common barrier among men in sub-Saharan Africa [20]. Improving self-efficacy through culturally tailored interventions, such as community-based health education and peer support programs, could encourage more men to undergo routine screening.

Furthermore, the study revealed that while many respondents recognised the benefits of CaP screening, this awareness did not necessarily translate into proactive health-seeking behaviour. This discrepancy is consistent with findings from previous research, which indicate that knowledge alone is insufficient to drive screening uptake unless coupled with strong cues to action, such as physician recommendations or community outreach programs [21]. The demographic characteristics of the study participants also provide insight into factors influencing late diagnosis. The majority of respondents had secondary education, and a significant proportion were employed in the private sector. Educational level has been identified as a key determinant of health literacy, influencing individuals’ ability to seek and utilise health information effectively [22]. Interventions aimed at increasing CaP awareness should therefore prioritise lower educated populations who may lack access to critical health information. According to empirical evidence, men of African ancestry are at higher risk or susceptible to CaP from 40 years of age [6]; this study focused on men aged 40 and above to promote early screening behaviours and reduce late-stage diagnoses of CaP. Recent findings from Nigeria [14] reveal that CaP is the second most common cancer among young men aged below 55 years, with a prevalence of 8.86%. These findings underscore the need to target younger age groups for early screening interventions to prevent disease progression.

## Conclusion

Using the HBM, this study was able to assess the predictors of late diagnosis of CaP among men of African ancestry receiving care at the urology clinic, Federal Medical Centre, Abeokuta.

In conclusion, as a low-hanging fruit, doctors and healthcare providers should prioritise encouraging adult males to engage in early and frequent CaP screenings, irrespective of whether they exhibit symptoms of the disease, as the majority of study participants responded that they had not gone for CaP screening because a doctor had not recommended it before; hence, this study recommends the development of a health policy that integrates CaP screening tests into routine health check-ups for all adult males. This approach is expected to promote early and regular screening, thereby reducing the prevalence of late-stage diagnoses of the disease. Therefore, it can be concluded from this study that, perceived susceptibility to CaP, the perceived severity of CaP, perceived benefits of CaP screening, self-efficacy and cues to action to CaP screening are all antecedent factors that predict late diagnosis of CaP among adult males.

## Recommendations for future study

While this study provided valuable insights into the predictors of late diagnosis of CaP among men of African ancestry, an additional factor that could further enrich the findings is understanding how a family member’s diagnosis of cancer influences perceptions and health-seeking behaviours. Future studies should consider including questions that explore this dimension to provide a more comprehensive understanding of familial influence on CaP awareness and screening behaviours.

## Limitations of the study

This study has some limitations. The sample population was limited to a specific geographic location, which may affect the generalisability of the findings. Additionally, self-reported data may be subject to recall and social desirability biases. Future research should consider larger, more diverse samples and explore additional socio-cultural factors influencing CaP screening behaviours.

## List of abbreviations

CaP, Prostate cancer; DRE, Digital rectum exam; HBM, Health belief model; PSA, Prostate specific antigen.

## Conflicts of interest

The authors declare that they have no conflicts of interest.

## Funding

This research received no external funding. The study was entirely self-funded by the authors.

## Ethical committee clearance

**Figure:**
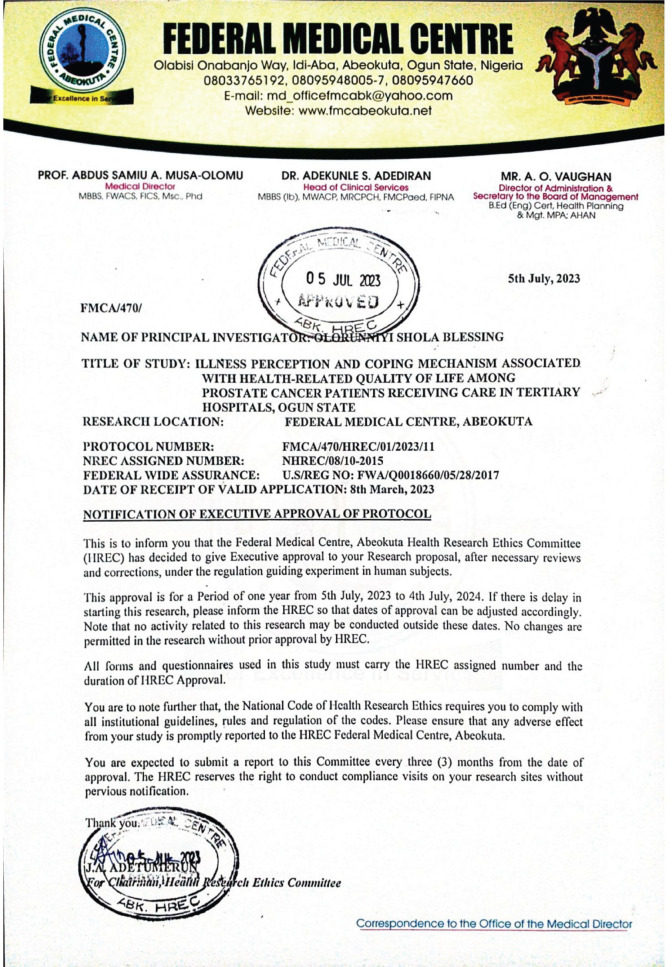


## Author contributions

Shola Blessing Olorunniyi: This author participated in the conceptualisation of the study, designed the study, ensured the acquisition, analysis and interpretation of data and wrote the manuscript.

Chidiebere Ndukwe Ogo: This author participated in the conceptualisation of the study and study design, supervised, coordinated the project and substantively revised the manuscript.

All authors have read and approved the final manuscript.

## Figures and Tables

**Table 1.** Demographic characteristics.

No. of respondents = 128		
Demographic variables	Frequency (*n*)	Percentage (%)
Age in years		
°40–50	80	62.5
°51–60	32	25.0
°61–70	16	12.5
Ethnicity		
°Yoruba	96	75.0
°Ibo	16	12.5
°Hausa	4	3.13
°Others	12	9.38
Educational level		
°Primary	12	9.38
°Secondary	100	78.1
°Tertiary	16	12.5
Employment status		
°Retired	32	25.0
°Civil servant	16	12.5
°Private sector	64	50.0
°Business owner	16	12.5

**Table 2.** Perceived susceptibility analysis table.

Items	SA (%)	A (%)	N (%)	D (%)	SD (%)	M	St	Perception
My chances of getting CaP are great	16 (12.5)	-	32 (25)	-	80 (62.5)	2	1.4	High
There is a good possibility that I will get CaP	-	16 (12.5)	32 (35)	16 (12.5)	64 (50)	2	1.1	High
I am not at risk for CaP	64 (50)	32 (25)	16 (12.5)	-	16 (12.5)	2	1.3	High
There is no chance that I will get CaP	80 (62.5)	32 (25)	16 (12.5)	-	-	1.5	0.7	Low

Note: SA **=** Strongly Agree; A = Agree; N = Neutral; D = Disagree; SD = Strongly Disagree; M = Mean; St = Standard deviation and the Weighted Average = 1.88

**Table 3.** Perceived severity analysis table.

Items	SA (%)	A (%)	N (%)	D (%)	SD (%)	M	St	Perception
If I had CaP my whole life would change	48 (37.5)	48 (37.5)	-	32 (25)	-	3.9	1.2	High
Getting CaP would significantly affect my life	32 (25)	48 (37.5)	16 (12.5)	32 (25)	-	3.6	1.1	High
I get very scared when I think of CaP	48 (37.5)	-	-	64 (50)	16 (12.5)	3	1.6	Low
Getting CaP would significantly affect my manhood	16 (12.5)	32 (25)	32 (25)	16 (12.5)	32 (25)	2.9	1.4	Low

Note: SA **=** Strongly Agree; A = Agree; N = Neutral; D = Disagree; SD = Strongly Disagree; M = Mean; St = Standard deviation and the Weighted Average = 3.35

**Table 4.** Perceived self-efficacy analysis table.

Items	VE (%)	E (%)	N (%)	D (%)	VD (%)	M	St	Perception
Making a decision about CaP screening is	16 (12.5)	-	16 (12.5)	96 (75)	-	2.5	1.0	High
Having a DRE every year is	-	32 (25)	-	48 (37.5)	48 (37.5)	2.1	1.2	Low
Giving my blood sample for serum PSA test every year is	-	32 (25)	-	80 (62.5)	16 (12.5)	2.4	0.9	High

Note: VE = Very Easy; E = Easy; N = Neutral; D = Difficult; VD = Very Difficult; M = Mean;

St = Standard deviation and the Ground mean/Weighted Average = 2.33

**Table 5.** Perceived benefits analysis table.

								
Items	SA (%)	A (%)	N (%)	D (%)	SD (%)	M	St	Perception
Preventing CaP through activities such as eating right, taking supplements and exercising will save my life	96 (75)	32 (25)	-	-	-	4.8	0.4	High
Preventing CaP through activities such as eating right, taking supplements, and exercising will help me to prevent other diseases.	80 (62.5)	48 (37.5)	-	-	-	4.6	0.5	High
Screening for CaP every year will allow me to detect the disease early and get appropriate treatment on time	64 (50)	48 (37.5)	16 (12.5)	-	-	4.38	0.7	Low
Getting screened for CaP every year will give me peace of mind	64 (50)	-	48 (37.5)	16 (12.5)	-	3.9	1.2	Low

Note: SA **=** Strongly Agree; A = Agree; N = Neutral; D = Disagree; SD = Strongly Disagree; M = Mean; St = Standard deviation and the Weighted Average = 4.41

**Table 6.** Perceived cues to action analysis table.

Items	SA (%)	A (%)	N (%)	D (%)	SD (%)	M	St	Perception
I do not get tested for CaP because my doctor has not recommended this test	48 (37.5)	32 (25)	16 (12.5)	16 (12.5)	16 (12.5)	3.6	1.4	High
I do not get tested for CaP because I forgot to do it.	16 (12.5)	-	-	80 (62.5)	32 (25)	2.1	1.2	Low
I do not get tested for CaP because I fear finding cancer.	-	16 (12.5)	16 (12.5)	64 (50)	32 (25)	2.1	0.9	Low
I have more important things to do that stops me from getting tested for CaP.	16 (12.5)	32 (25)	16 (12.5)	32 (25)	32 (25)	2.8	1.4	High
I am too busy to be tested for CaP	-	16 (12.5)	32 (25)	48 (37.5)	32 (25)	2.3	0.9	Low
I am embarrassed to be tested for CaP	-	64 (50)	-	16 (12.5)	48 (37.5)	2.6	1.4	High
I do not get tested for CaP because I cannot afford the test	-	32 (25)	16 (12.5)	48 (37.5)	32 (25)	2.4	1.1	Low
I do not get tested for CaP because I am afraid of experiencing pain and discomfort	-	64 (50)	-	32 (25)	32 (25)	2.8	1.3	High
I do not get tested for CaP because I am not of screening age	-	48 (37.5)	-	32 (25)	48 (37.5)	2.4	1.3	Low

Note: SA = Strongly Agree; A = Agree; N = Neutral; D = Disagree; SD = Strongly Disagree; M = Mean; St = Standard deviation and the Weighted Average = 2.56
